# Accuracy of a Nutrient Database in Estimating the Dietary Phosphorus-to-Protein Ratio and Using a Boiling Method in Low-Phosphate Hospital Diets

**DOI:** 10.1038/s41598-018-33657-8

**Published:** 2018-10-15

**Authors:** Wan-Chuan Tsai, Yu-Sen Peng, Hon-Yen Wu, Shih-Ping Hsu, Yen-Ling Chiu, Lie-Chuan Liu, Shu-Min Tsai, Kuo-Liong Chien

**Affiliations:** 1 Department of Internal Medicine, Far Eastern Memorial Hospital, New Taipei City, Taiwan; 2 Institute of Epidemiology and Preventive Medicine, College of Public Health, National Taiwan University, Taipei City, Taiwan; 3 Oriental Institute of Technology, New Taipei City, Taiwan; 4 Department of Internal Medicine, National Taiwan University Hospital and College of Medicine, Taipei City, Taiwan; 5 Faculty of Medicine, School of Medicine, National Yang-Ming University, Taipei City, Taiwan; 6 Graduate Program in Biomedical Informatics, Yuan Ze University, Taoyuan City, Taiwan; 7 Dietary Department, Far Eastern Memorial Hospital, New Taipei City, Taiwan

## Abstract

The use of the dietary phosphorus-to-protein ratio (PPR) to reduce dietary phosphorus while maintaining protein intake is valuable for nutritional management in the dialysis population, but the actual PPR values in hospital meals have not been determined. We aimed to determine the accuracy of a nutrient database for estimating the PPR in low-phosphate hospital diets compared with the accuracy of chemical analysis and produce hospital diets with low-phosphate content by boiling meat for 30 minutes before cooking. The phosphorus and protein content of 26 cooked dishes selected from the hospital menu was estimated using a food composition table (FCT) and sent for chemical analysis. Comparisons of FCT-based estimated values with measured values for every 100 g of tested foods revealed an overestimation for the PPR both in plant-based dishes (mean difference ± SD, 4.1 ± 14.6 mg/g, *P* = 0.06), and in meats (2.1 ± 2.3 mg/g, *P* = 0.06). By boiling meats, we crafted diets with PPR as low as 8 mg/g. Caution should be exercised in estimating the PPR using a FCT in hospital diets and boiling should be used to prepare hospital meals. Such diets will be promoted for dialysis patients in both inpatient and outpatient settings.

## Introduction

Hyperphosphatemia is common among dialysis patients, and dietary phosphorus restriction is an effective way to control serum phosphorus level^1,2^. Adequate dietary protein intake is of utmost importance for patients undergoing dialysis^3^. However, the inherent relationship between phosphorus and protein content in foods makes it difficult to plan a low-phosphate diet without restrictions on protein intake^4,5^. Kidney Disease Outcomes Quality Initiative (K/DOQI) clinical practice guidelines^6^ suggested the dietary phosphorus-to-protein ratio (PPR) as a more suitable measurement than the phosphorus content of food alone to ensure provision of sufficient protein content with the lowest possible phosphorus content, and the PPR has been used in the nutrient database for a Spanish population^7^. The PPR is a valuable metric for dietary phosphorus management^8^, and a high PPR value is independently associated with an increased risk of death in hemodialysis patients^9^.

There is growing evidence that eating during hemodialysis treatment can improve nutritional status, quality of life and survival^10,11^. Low-phosphate hospital diet is one of the useful candidates to provide foods during hemodialysis treatment. However, the amount of phosphorus and the PPR of currently used low-phosphate meals for dialysis patients in our hospital are estimated based on a food composition table (FCT)^12^, and their actual content has not been determined. The FCT is essential for estimating nutrient intake in clinical practice, but estimation of dietary phosphorus intake using the FCT has several limitations^13^. Importantly, data from the FCT are restricted to raw foods and have little information regarding the changes in nutrients after cooking; thus, mineral changes during the cooking process cannot be accurately ascertained^14^. In addition, the phosphorus content of meat is affected by the cooking time, boiling method and extent of meat shredding, and this information is not available in a nutrient database^15,16^. Moreover, the relationship between FCT-based estimates of dietary phosphorus intake and direct chemical analysis has been studied by several investigators with conflicting results^17–20^, which are due in part to differences in dietary phosphorus sources, food additives, and dietary assessment methods. Furthermore, there is little literature concerning the association between FCT-based estimation and direct measurement of PPR content, and meat boiling has not been utilized in low-phosphate hospital diets.

The aims of this study were to determine the accuracy of a Taiwanese nutrient database for estimating the PPR in low-phosphate hospital diets compared with the accuracy of direct measurement and produce low-phosphate hospital diets using demineralization of meat by a boiling method. In the future, these low-phosphate hospital diets can be delivered to dialysis patients to control serum phosphate level.

## Results

### Accuracy of the nutrient database

Table 1 shows the comparison of FCT-based E with M of the PPR, phosphorus, protein, and calcium for a total of 20 tested foods, and there is a substantial variation in differences (E-M) among study foods. Table 2A summarizes the comparison of E with M of nutrients for plant-based dishes (N = 14), including 2 fruits, 4 grains, 4 side dishes, and 4 vegetables. The mean value of difference for the PPR was 4.1 ± 14.6 mg/g (*P* = 0.06), indicating that FCT estimation overestimated the actual content. However, the differences for phosphorus, protein and calcium did not achieve statistical significance. In regression analysis, the PPR exhibited poor agreement between E and M due to the highest intercept value with the slope (β) less than 0.5. However, phosphorus, protein and calcium showed good agreement as the values of the intercept were relatively small, and the slopes were close to 1.0. For phosphorus, protein and calcium, there was a strong linear correlation and excellent reliability between E and M for plant-based dishes because of the high values of r and ICC, i.e., 0.96 and 0.96 for phosphorus, 0.95 and 0.90 for protein, and 0.96 and 0.96 for calcium. In contrast, the PPR had a poor linear correlation (r = 0.37, *P* = 0.19) and reliability (ICC = 0.36).

**Table 1:** Estimated (E) and Measured (M) Values of Nutrients for a Total of 20 Food Items and their Difference (E - M).

Category	Food items	Phosphorus (mg/100 g)			Calcium (mg/100 g)			Protein (g/100 g)			Phosphorus/Protein Ratio (mg/g)		
		E	M	E - M	E	M	E - M	E	M	E - M	E	M	E - M
Fruit	N = 2												
	Orange	20.8	20.7	0.1	28.4	37.6	−9.2	0.8	1.0	−0.2	26.1	20.7	5.4
	Apple	9.5	7.9	1.6	3.9	6.8	−2.9	0.2	0.5	−0.3	49.2	15.8	33.4
Grain	N = 4												
	Steamed bread (mantou)	58.0	62.9	−4.9	21.0	10.9	10.1	8.1	9.1	−1.0	7.2	6.9	0.2
	Fried bean thread noodles with celery cabbage	13.8	17.9	−4.1	4.2	10.2	−6.0	0.2	0.6	−0.4	59.0	29.8	29.1
	Steamed rice	39.3	24.3	15.0	1.4	2.5	−1.1	3.1	3.0	0.1	12.7	8.1	4.6
	Fried rice stick noodle with celery cabbage	15.5	31.6	−16.1	12.9	10.8	2.1	0.7	0.6	0.1	22.4	52.7	−30.3
Meat	N = 6												
	Grilled pork lean meat with white sesame seeds	204.8	189.0	15.8	5.0	9.0	−4.0	19.9	21.6	−1.7	10.3	8.8	1.5
	Sliced boiled pork with garlic sauce	207.8	86.5	121.3	3.3	8.0	−4.7	20.5	22.5	−2.0	10.1	3.8	6.3
	Black pepper pork foreshank	188.1	181.0	7.1	6.8	10.4	−3.6	19.7	24.5	−4.8	9.5	7.4	2.2
	Poached chicken with scallion oil	142.5	149.0	−6.5	10.9	15.2	−4.3	17.5	20.9	−3.4	8.2	7.1	1.0
	Grilled salmon with black sesame seeds	217.1	252.0	−34.9	10.7	44.1	−33.4	22.3	24.5	−2.2	9.7	10.3	−0.6
	Braised chicken cutlets	142.8	145.0	−2.2	5.9	9.8	−3.9	15.4	20.2	−4.8	9.3	7.2	2.1
Side dish	N = 4												
	Braised tofu with black fungus	79.9	72.3	7.6	97.7	64.4	33.3	5.8	5.7	0.1	13.8	12.7	1.1
	Stir-fried cucumber and bean curd skin	172.9	192.0	−19.1	137.2	175.0	−37.8	8.8	13.8	−5.0	19.6	13.9	5.6
	Stir-fried sweet pepper and bean curd noodles	132.7	167.0	−34.3	128.0	151.0	−23.0	8.5	12.8	−4.3	15.7	13.0	2.7
	Stir-fried Chinese chive flower and soybean curd	143.1	122.0	21.1	128.3	134.0	−5.7	9.1	8	1.1	15.7	15.3	0.4
Vegetable	N = 4												
	Fried sweet potato leaves	41.0	29.0	12.0	99.2	78.7	20.5	3.0	2.6	0.4	13.8	11.2	2.7
	Fried green bean	34.6	31.7	2.9	36.8	41.4	−4.6	1.6	1.8	−0.2	21.3	17.6	3.7
	Fried broccoli	38.7	63.3	−24.6	35.7	33.8	1.9	2.4	3.0	−0.6	16.3	21.1	−4.8
	Fried pak-choi	28.4	28.3	0.1	97.3	94.9	2.4	1.2	1.4	−0.2	24.3	20.2	4.0

**Table 2:** Comparison of Estimated Values with Measured Values of Nutrients for Plant-based Dishes (A) and Meats (B).

Dietary elements	Estimated (E)	Measured (M)	Difference (E - M)	*P* value*	Regression analysis^†^		Correlation coefficient		Intraclass correlation coefficient (ICC)
	Mean ± SD	Mean ± SD	Mean ± SD		Intercept (α)	Regression coefficient (β)	r	*P* value	
(**A**) Plant-based Dishes (N = 14)									
Phosphorus/Protein Ratio (mg/g)	22.6 ± 14.4	18.5 ± 11.5	4.1 ± 14.6	0.06	11.7	0.3	0.37	0.19	0.36
Phosphorus (mg/100 g)	59.2 ± 52.9	62.2 ± 58.0	−3.1 ± 15.7	0.71	−0.3	1.1	0.96	<0.001	0.96
Protein (g/100 g)	3.8 ± 3.5	4.6 ± 4.6	−0.7 ± 1.7	0.12	−0.2	1.2	0.95	<0.001	0.90
Calcium (mg/100 g)	59.4 ± 52.0	60.9 ± 57.9	−1.4 ± 17.0	0.58	−2.5	1.1	0.96	<0.001	0.96
(**B**) Meats (N = 6)									
Phosphorus/Protein Ratio (mg/g)	9.5 ± 0.8	7.4 ± 2.1	2.1 ± 2.3	0.06	8.0	−0.1	−0.02	0.97	0
Phosphorus (mg/100 g)	183.9 ± 33.3	167.1 ± 55.1	16.8 ± 54.0	0.69	65.2	0.6	0.33	0.52	0.33
Protein (g/100 g)	19.2 ± 2.4	22.4 ± 1.8	−3.1 ± 1.4	0.03	10.5	0.6	0.82	0.04	0.18
Calcium (mg/100 g)	7.1 ± 3.1	16.1 ± 13.9	−9.0 ± 12.0	0.03	−6.7	3.2	0.71	0.11	0.14

Notes. *Wilcoxon signed-rank test. ^†^α and β are parameters of a regression line: y = α + βx, where y and x are measured and estimated values of the dietary elements, respectively.

Abbreviations. SD, standard deviation.

Table 2B summarizes the comparison of E with M of nutrients for meats (N = 6). The mean value of difference for the PPR was 2.1 ± 2.3 mg/g (*P* = 0.06), indicating that FCT estimation overestimated the actual content. However, the difference for phosphorus did not reach statistical significance. For protein and calcium, the differences were significantly less than zero (both *P* = 0.03), meaning that FCT estimation underestimated the direct measurement. In regression analysis, the PPR showed poor agreement between E and M due to the slope (β) less than 0.5. In contrast, phosphorus, protein and calcium exhibited good agreement as the slopes were greater than 0.5. Among nutrients, only protein had a strong linear correlation between E and M (r = 0.82, *P* = 0.04). All nutrients showed poor reliability between E and M with a range of ICC between 0 and 0.33.

**Figure 1:**
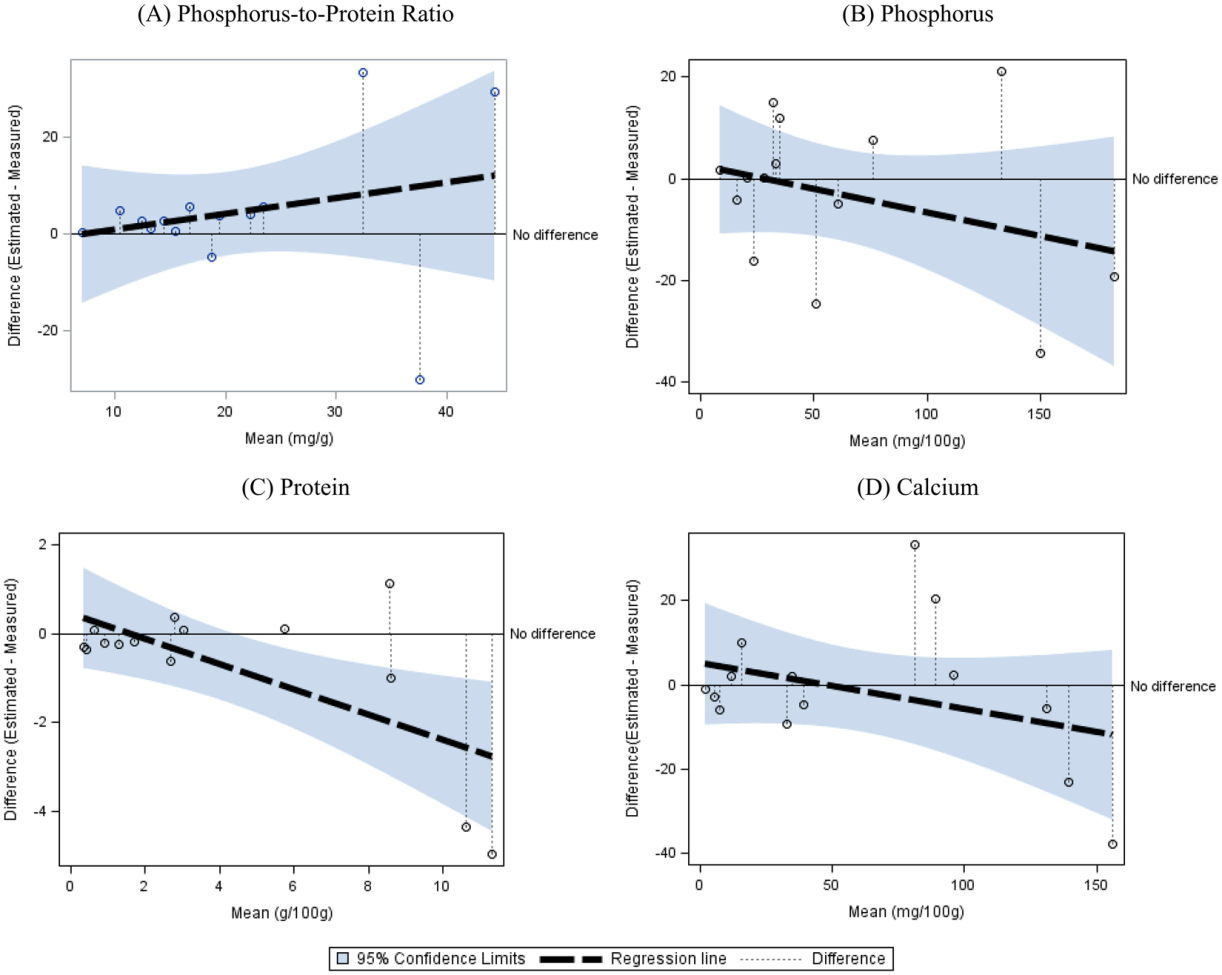
Bland-Altman Difference Plot for Plant-based Dishes Assessing the Agreement between Estimated and Measured Values for Nutrients including (**A**) Phosphorus-to-Protein Ratio, (**B**) Phosphorus, (**C**) Protein and (**D**) Calcium. The “No difference” line reflects perfect agreement between estimated and measured values. The dashed line depicts the regression line demonstrating differences between estimated and measured values against their mean, and the upper and lower lines surrounding the dashed line are 95% confidence limits. The vertical dotted line indicates the difference between estimated and measured values for each food item. If the regression line appears above the “No difference” line, the food-table-based estimation overestimates the actual content by direct measurement and vice versa.

**Figure 2:**
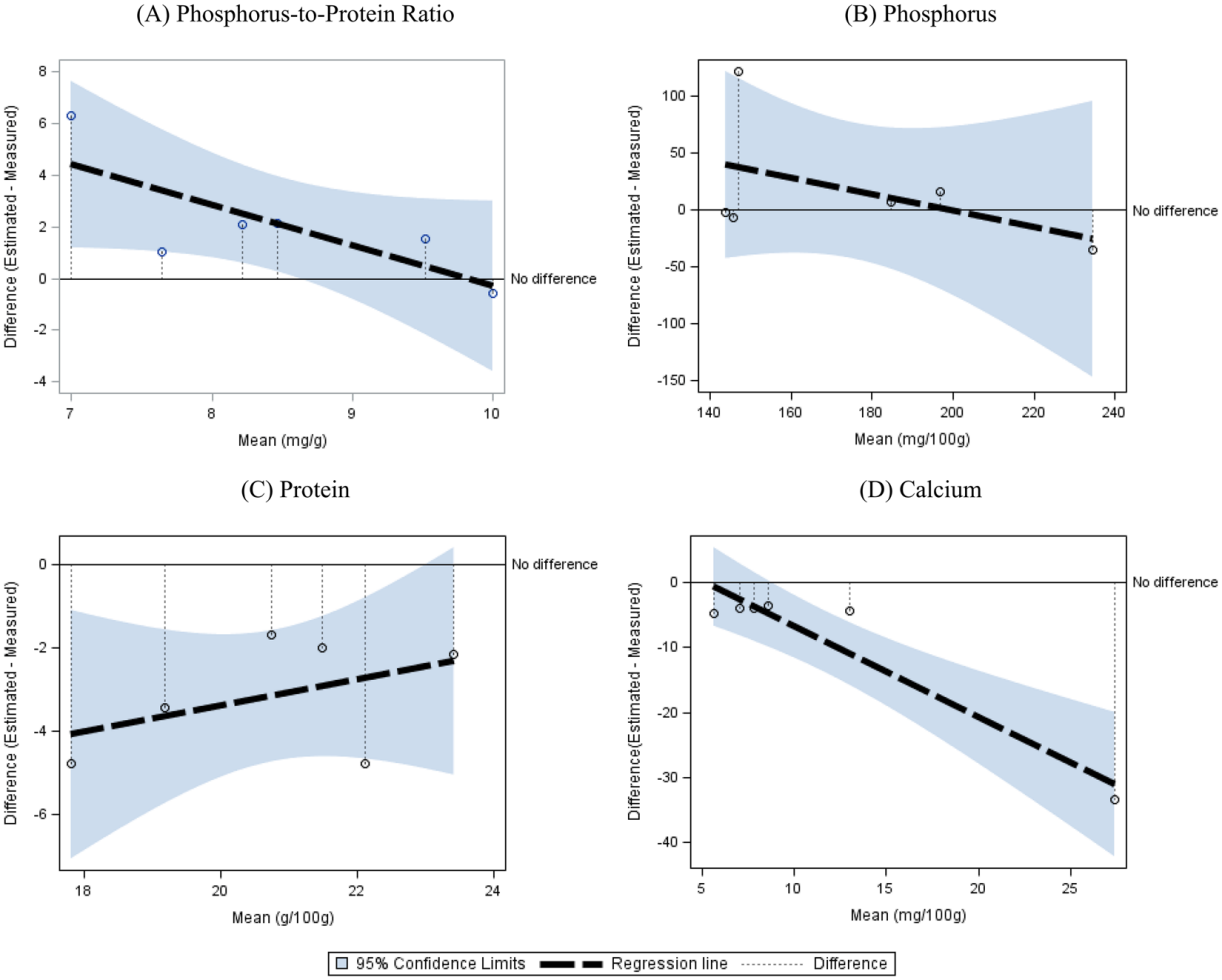
Bland-Altman Difference Plot for Meats Assessing the Agreement between Estimated and Measured Values for Nutrients including (**A**) Phosphorus-to-Protein Ratio, (**B**) Phosphorus, (**C**) Protein and (**D**) Calcium. The “No difference” line reflects perfect agreement between estimated and measured values. The dashed line depicts the regression line demonstrating differences between estimated and measured values against their mean, and the upper and lower lines surrounding the dashed line are 95% confidence limits. The vertical dotted line indicates the difference between estimated and measured values for each food item. If the regression line appears above the “No difference” line, the food-table-based estimation overestimates the actual content by direct measurement and vice versa.

Figure 1 shows Bland-Altman difference plots for plant-based dishes assessing the agreement between E and M for the PPR, phosphorus, protein and calcium. In Fig. 1A, FCT estimation overestimated the actual value of the PPR because the regression line appears above the “No difference” line, and overestimation was magnified as the PPR value of a given food increased. In contrast, in Fig. 1B–D, the regression lines for phosphorus, protein and calcium are located below the “No difference” line, demonstrating that FCT estimation underestimated the actual content. Figure 2 shows Bland-Altman difference plots for meats assessing the agreement between E and M for the PPR, phosphorus, protein and calcium. In Fig. 2A, FCT estimation overestimated the actual value of the PPR because the regression line appears above the “No difference” line. For phosphorus, the regression line is distributed across the “No difference” line, providing a variation in FCT estimation depend on the tested foods (Fig. 2B). In contrast, in Fig. 2C,D, the regression lines for protein and calcium are located below the “No difference” line, revealing that FCT estimation underestimated the actual protein and calcium contents.

### Effect of boiling before cooking on changes in nutrients in meats

The detailed data for the nutrient content of 12 foods, including 6 meats prepared without an extra boiling for 30 minutes and 6 meats that underwent extra boiling for 30 minutes before cooking, are provided in Table S1. Table 3 summarizes and compares the changes in nutrients in meats prepared with and without boiling for 30 minutes. With cooking, the PPR and the amount of phosphorus in meats decreased, and the amount of protein and calcium increased. After meats were boiled for 30 minutes, the PPR and the amount of phosphorus decreased further, and the increased amount of protein and calcium became apparent. As shown in Table 3, the mean difference between meats prepared with and without boiling for 30 minutes reached statistical significance for phosphorus (*P* = 0.03) and trended toward significance for PPR (*P* = 0.06) and protein (*P* = 0.09); however, there was no difference for calcium (*P* = 0.44).

**Table 3:** Comparison of Estimated Values with Measured Values of Nutrients for Meats Prepared with and without Boiling for 30 Minutes.

Dietary elements	N = 6 meats without an extra boiling			N = 6 meats with boiling for 30 minutes before cooking			Comparison	
	Estimated (E)	Measured (M)	Difference (E - M)	Estimated (E)	Measured (M)	Difference (E - M)	MD	*P* value*
Phosphorus/Protein Ratio (mg/g)	9.5 ± 0.8	7.4 ± 2.1	2.1 ± 2.3	9.5 ± 0.8	6.0 ± 1.7	3.5 ± 2.2	−1.5	0.06
Phosphorus (mg/100 g)	183.9 ± 33.3	167.1 ± 55.1	16.8 ± 54.0	183.6 ± 33.2	145.5 ± 44.5	38.2 ± 52.2	−21.4	0.03
Protein (g/100 g)	19.2 ± 2.4	22.4 ± 1.8	−3.1 ± 1.4	19.2 ± 2.4	24.5 ± 3.7	−5.3 ± 2.0	2.1	0.09
Calcium (mg/100 g)	7.1 ± 3.1	16.1 ± 14.0	−9.0 ± 12.0	7.1 ± 3.0	21.4 ± 26.3	−14.4 ± 24.6	5.4	0.44

Notes. Continuous data are provided as the mean ± standard deviation. *Wilcoxon signed-rank test.

Abbreviations. MD, mean difference.

### Production of low-phosphate hospital meals with the boiling method for clinical use

Using the measured values of the PPR, we crafted a low-phosphate hospital diet that can be provided to dialysis patients as part of the management of hyperphosphatemia in the future. The actual PPR values of ingredients in low-phosphate hospital diets are provided in Table S2. The low-phosphate hospital diet comprised 3 meals a day. Breakfast included steamed bread and a mixture of nutritional supplements; each lunch and dinner included 1 meat, 1 side dish, 1 vegetable, 1 grain and 1 fruit. Due to the significant phosphate-lowering effect of the boiling method in meats, we prepared meats with boiling before cooking. Table 4 shows the daily nutrient content of low-phosphate hospital diets. In consideration of common ranges of body size in our dialysis population, low-phosphate hospital diets were categorized according to the corresponding body weight. To reduce dietary phosphate while maintaining protein intake, we produced two different PPR values, 8 mg/g and 10 mg/g.

**Table 4:** Low-Phosphate Hospital Diets Categorized According to Body Weight and the Dietary Phosphorus-to-Protein Ratio.

Category	Body Size (kg)	Calorie (kcal/day)	Protein (g/day)	Phosphorus (mg/day)	Phosphorus/Protein Ratio (mg/g)
PPR = 8 mg/g					
	45	1368.1	56.5	447.9	7.9
	50	1502.0	61.6	473.1	7.7
	55	1662.5	70.5	537.8	7.6
	60	1807.8	77.5	575.0	7.4
	65	1949.5	80.0	641.1	8.0
PPR = 10 mg/g					
	45	1340.6	58.8	585.2	10.0
	50	1492.5	63.0	620.8	9.9
	55	1609.4	66.3	653.7	9.9
	60	1768.4	74.0	741.8	10.0
	65	1937.0	78.7	813.7	10.3

Abbreviations. PPR, Phosphorus-to-Protein Ratio.

## Discussion

This is the first study to assess the accuracy of nutrient database in estimating the dietary PPR and produce low-phosphate hospital meals using boiling meats with PPR as low as 8 mg/g. In our study, FCT-based estimation for both plant-based dishes and meats overestimated the actual value of the PPR in natural foods chosen from a low-phosphate hospital menu. The results from the Bland-Altman difference plot, regression analysis, correlation analysis and intraclass correlation coefficient concordantly showed that FCT-based estimation and direct measurement exhibited poor agreement for the PPR. In contrast, FCT-based estimation was consistent with direct measurement for phosphorus; therefore, estimation using the FCT for phosphorus content may be reasonably accurate. We also demonstrated that a pre-cooking process with boiling reduced phosphorus content and PPR value of meats. Using natural food items and taking the phosphate-lowering effect of the boiling method in meats into account, it is feasible for dietitians to craft low-phosphate hospital diets with PPR values as low as 8 mg/g for dialysis patients. In accordance with the strategy like “Eating during hemodialysis treatment”, we are going to promote such diets for dialysis patients to control serum phosphate level in both inpatient and outpatient settings.

The widespread notion that the FCT underestimates actual phosphorus content in foods was largely based on studies conducted on phosphate additives^17,18^. Our study foods were selected from a routine hospital menu and had a comparable result for phosphorus content estimated using the FCT or analyzed by direct measurement. These comparable results were due to the foods used in our hospital meals, which were natural, free of additives, and had data on the nutrient database; moreover, the seasonings used in the cooking process were estimated. Notably, the difference between FCT-based estimation and direct measurement for phosphorus was inconsistent among the study foods; specifically, the FCT overestimated the measured value in 11 of 20 foods (Table 1). Differences in food preparation and cooking methods used in our study foods possibly caused the varying results between FCT estimation and direct measurement^21,22^. Loss of phosphorus during cooking might partially explain why the estimated value overestimated the directly measured one^14^.

Even though dietary phosphorus restriction is of great importance in the management of hyperphosphatemia, caution should be exercised to avoid malnutrition. Controlling serum phosphorus levels by imposing dietary protein restriction may have a poor outcome in patients undergoing dialysis^23^. Foods with a PPR between 10–12 mg/g are recommended to limit phosphorus intake while providing adequate protein for dialysis patients^6^. In our study, the nutrient database deviated from the actual amount of PPR by 4.1 mg/g for plant-based dishes and 2.1 mg/g for meats, highlighting the importance of more accurate estimation of dietary intake by the FCT in terms of the PPR. These results were similar to a previous study^15^ in which the PPR in foods was significantly lowered by cooking. The FCT-based overestimation for the PPR possibly occurred because of a joint effect from an acceptable deviation for phosphorus estimation and a significant underestimation of protein content. We recognized the PPR as a useful marker to choose appropriate foods and prepare low-phosphate hospital diets. Based on the results of our study, we crafted a low-phosphate daily hospital diet with a PPR value of 8 mg/g, which is less than the value recommended by K/DOQI guidelines^6^. These findings demonstrate that it is feasible for dietitians to produce low-phosphate diets for clinical use.

The phosphorus content in meats was significantly lower after boiling for 30 minutes before cooking than after usual cooking. In a study by Cupisti *et al*.^15^, the amount of phosphorus loss increased with boiling times of 10, 20, and 30 minutes and the expected amount of phosphorus loss were detected in the boiling fluid. Meat size is another factor that determines phosphorus loss after boiling^16^. In our study, loss of phosphorus after cooking was significant for sliced meats, i.e., sliced boiled pork with garlic sauce, in which phosphorus within the cell and cell membrane was more vulnerable to loss than that in dishes with larger meat size, i.e., braised chicken cutlets, poached chicken with scallion oil or black pepper foreshank.

FCT-based estimation underestimated actual calcium and protein content, especially in meats. The calcium content of tap water is high, and increased amounts of calcium in study foods after cooking can be caused by contamination of tap water^14^. Our study indicated that such contamination may be magnified as the calcium content of study foods increased further after boiling in tap water for 30 minutes as shown in Table 3. We noticed that the mean difference in g/100 g for protein in each cooked food category was consistently less than zero; the values were −0.3 for grains, −3.1 for meats, −2.0 for side dishes and −0.2 for vegetables, as presented in Table 1. Thus, depending on the type of food consumed, FCT-based estimated values were less than the measured values for protein by 10% to 21%. In addition, there was a strong linear correlation (r = 0.95, *P* < 0.001 in plant-based dishes; r = 0.82, *P* = 0.04 in meats) between FCT-based estimation and direct measurement for protein, indicating consistent changes in protein content in the same direction, i.e., higher protein content after cooking among cooked foods. Furthermore, prolonged boiling in meats tended to make the difference significant (*P* = 0.09, Table 3). Similar to our finding, Cupisti and colleagues demonstrated that the percentage of protein content in beef or chicken breast progressively increased after 10, 20, and 30 minutes of boiling^15^. The consistency in the increased amount of protein after boiling suggested that the foods became condensed after water loss during the cooking process and retaining protein in cells. The data regarding changes in protein content during the cooking process are not available in the nutrient database, preventing the accurate estimation of protein intake.

Our study had a few limitations. First, the number of foods included in our study is small, especially for foods used in the boiling method. We selected the most commonly consumed dishes from the hospital menu and did not include foods with seasonal restrictions, which narrowed the range of the candidate foods for the study. However, the foods included in our study were favored by our patients and were available all year long. To fulfill the diversity of food preference among patients, more food items should be studied for nutrient analysis in the future. Second, we mainly included food items which had data on the Taiwanese nutrient database. Subsequently, the findings of our study were restricted to the available food in the nutrient database; thus, external generalization should be cautioned. Third, the change in the amount of water due to vaporization and changes in minerals in cooking liquid were not measured. However, such information has been assessed in previous studies^14^. The currently available nutrient database lacked such data, and dietitians face similar problems in clinical practice. To minimize the bias and reflect clinical practice, we compared estimated values with measured values in 100 g of each nutrient, which enhanced the efficiency of comparison.

In conclusion, estimation of nutrients using the FCT in natural foods from a routine hospital menu may be acceptable for phosphorus, but caution should be exercised in estimation of the PPR. A pre-cooking process with boiling for 30 minutes in meats may decrease the amount of phosphorus and PPR and should be used to prepare hospital meals. Such low-phosphate hospital diets should be promoted for dialysis patients to provide an adequate nutrition support with the lowest phosphate content in both inpatient and outpatient settings.

## Methods

### Characteristics of the study food sources

To enhance the representativeness of hospital diets, we selected highly consumed dishes from the hospital menu. Since food additives include readily absorbable inorganic phosphorus, only natural food sources were chosen. Seasonal food items were not selected in the hope of continuation of the meal supply in our hospital throughout the year. All study food items had the following unique characteristics including: (1) Using locally produced raw materials. (2) Meeting healthy and safety requirements. (3) Complying with national quality standards.

### Preparation of study foods

The dietitian selected a total of 20 dishes from our hospital menu, including 2 fruits, 4 grains, 6 meats, 4 side dishes, and 4 vegetables. With different combinations, these dishes are used to make 3 meals a day. For example, breakfast includes steamed bread with a drink and each of lunch or dinner includes 1 meat, 1 side dish, 1 vegetable, 1 grain and 1 fruit. We prepared 6 additional meats from the selected 20 dishes in different ways to assess the effect of the boiling method in reducing phosphorus amounts. These 6 meats were boiled in water for 30 minutes, and the water was discarded before cooking. With the food hygiene practice using Hazard Analysis and Critical Control Points (HACCP) system^24^, food preparation and cooking were performed by an experienced cook in our hospital kitchen in the dietary department. Our hospital kitchen received and renewed HACCP certification periodically.

### Improving the palatability of the boiled meats

To improve the taste of meats with boiling method, the cook had adjusted the subsequent preparation and cooking methods. First, shortening the subsequent cooking time after boiled meat to make meat tender, for example, the oven roasting time was reduced from 50 to 15 minutes for black pepper pork foreshank after boiling in water for 30 minutes. Second, thickening the meat with the tapioca to increase smoothness, as in the poached chicken with scallion oil. Third, making a juicier dish by drizzling the sauce over the meat, as in the sliced boiled pork with garlic sauce. Finally, special flesh spices such as green onion, garlic, five spices powder or black pepper and rice-wine were used in boiled meats to add flavor. The detailed description for ingredients, preparation and cooking methods of the study menu are provided in Table S3.

### Estimation of nutrient content in low-phosphate hospital meals

To improve estimates, we restricted our selection to foods that already had data on the FCT^12^. The phosphorus, calcium and protein content of dishes was assessed by using the latest available version of the FCT. We then calculated the PPR (mg/g) for each dish. Estimation of nutrients involved the amount of oil, soy sauce, salt, sugar, pepper, potato starch, garlic, and allspice used. The average value for a given food item was preferred if provided in the FCT.

### Analysis and measurement of nutrients in low-phosphate hospital meals

A total of 26 cooked dishes were manually separated in plastic bags and transported to a qualified laboratory for analysis of phosphorus and protein. Calcium was also measured as a control group. Laboratory technicians were blinded to the nutritional content assessed by the nutrient database. With reference to Association of Official Analytical Communities (AOAC) Official Method 984.27^25^, phosphorus and calcium were determined by inductively coupled plasma-optical emission spectrometer (ICP-OES) analysis with a detection limit of 0.1 mg/L. In brief, the sample weights were obtained, the edible portions of samples were ashed at a high temperature, digested in nitric acid, and subjected to ICP-OES to determine their actual phosphorus and calcium contents. The amount of protein was measured with reference to Chinese National Standards (CNS) 5035^26^.

### Production of low-phosphate hospital meals with meat boiling

Based on the data from direct measurement of 26 tested foods, we crafted daily hospital diets with low-phosphate content, and each diet included 3 meals a day to fulfill the following criteria suggested by K/DOQI clinical practice guidelines^6,27^, including daily energy intake greater than 30 kcal/kg body weight, high-protein diet greater than 1.2 g/kg/day, phosphorus content less than 800 mg/day, and PPR less than 10 mg/g. With reduction of phosphorus content by demineralization of meat with the boiling method, we produced low-phosphate hospital diets with two different PPR values of 8 mg/g and 10 mg/g.

### Statistics

Continuous measures were summarized by means (±standard deviations) and categorical variables by counts. First, we assessed the accuracy of the nutrient database by comparing FCT-based estimated values (E) with corresponding measured values (M) of 20 tested foods for each nutrient. As the FCT estimated the amount of nutrients in values per 100 g of nutrients, the results of direct measurement were expressed as mg/100 g for phosphorus and calcium and g/100 g for protein. We calculated the PPR of tested foods in mg/g, which can be used as a marker for selection of an appropriate low-phosphorus and high-protein food. We then calculated the difference (E minus M) for each nutrient. If the value of the difference was less than 0, the FCT-based estimation underestimated the direct measurement and vice versa. According to difference in the amount of protein and phosphate bioavailability, the tested foods were categorized into plant-based dishes and meats, and data were presented separately. To graphically assess the agreement between FCT-based estimation and direct measurement for each nutrient, we illustrated Bland-Altman difference plots in which differences were plotted against their mean values^28^. Wilcoxon signed-rank tests were performed to examine the difference between paired measurements. The correlation between the E and M of nutrients was further examined by the following methods: linear regression models, correlation coefficients (r), and intraclass correlation coefficients (ICC). Second, we separately calculated the difference for 6 meats prepared with usual cooking and another 6 identical meats, which were boiled for 30 minutes before cooking, to investigate the effect of boiling before cooking on changes in nutrients. To assess whether changes in nutrients differed between the two different kinds of cooking, we performed Wilcoxon signed-rank tests. All the analyses and graphs were performed with SAS software (version 9.4; SAS Institute Inc., Cary, NC, USA).

## Electronic supplementary material

Dataset 1

## Data Availability

The datasets analyzed during the current study are available from the corresponding author on reasonable request.
