# Supplementary material for: Accuracy of a Nutrient Database in Estimating the Dietary Phosphorus-to-Protein Ratio and Using a Boiling Method in Low-Phosphate Hospital Diets
Source: Sci Rep. 2018 Oct 15;8:15246. doi: 10.1038/s41598-018-33657-8 (PMC6189135; doi:10.1038/s41598-018-33657-8)
Supplement: Supplementary file 1 — Dataset 1 [file 41598_2018_33657_MOESM1_ESM.pdf]

SUPPLEMENTARY DATA

Accuracy of a Nutrient Database in Estimating the Phosphorus-to-Protein Ratio and Using a Boiling Method in Low-Phosphate Hospital Diets

Wan-Chuan Tsai, Yu-Sen Peng, Hon-Yen Wu, Shih-Ping Hsu, Yen-Ling Chiu, Lie-Chuan Liu, Shu-Min Tsai,* Kuo-Liong Chien*

Table S1. Estimated (E) and Measured (M) Values of Nutrients for Meats Prepared with and without Boiling for 30 Minutes and their Differences (E - M)
Table S2. Phosphorus-to-Protein Ratio of Ingredients in a Daily Menu of Low-Phosphate Hospital Diets
Table S3. Ingredients, Preparation and Cooking Methods of the Study Menu

Table S1. Estimated (E) and Measured (M) Values of Nutrients for Meats Prepared with and without Boiling for 30 Minutes and their Differences (E - M)

Group	Food items	Phosphorus (mg/100 g)			Calcium (mg/100 g)			Protein (g/100 g)			Phosphorus/Protein Ratio (mg/g)		
		E	M	E - M	E	M	E - M	E	M	E - M	E	M	E - M
N = 6 meats with no extra boiling													
	Grilled pork lean meat with white sesame seeds	204.8	189.0	15.8	5.0	9.0	-4.0	19.9	21.6	-1.7	10.3	8.8	1.5
	Sliced boiled pork with garlic sauce	207.8	86.5	121.3	3.3	8.0	-4.7	20.5	22.5	-2.0	10.1	3.8	6.3
	Black pepper pork foreshank	188.1	181.0	7.1	6.8	10.4	-3.6	19.7	24.5	-4.8	9.5	7.4	2.2
	Poached chicken with scallion oil	142.5	149.0	-6.5	10.9	15.2	-4.3	17.5	20.9	-3.4	8.2	7.1	1.0
	Grilled salmon with black sesame seeds	217.1	252.0	-34.9	10.7	44.1	-33.4	22.3	24.5	-2.2	9.7	10.3	-0.6
	Braised chicken cutlets	142.8	145.0	-2.2	5.9	9.8	-3.9	15.4	20.2	-4.8	9.3	7.2	2.1
N = 6 meats that have been boiled for 30 minutes													
	Grilled pork lean meat with white sesame seeds	204.8	130.0	74.8	5.0	11.5	-6.5	19.9	26.2	-6.3	10.3	5.0	5.3
	Sliced boiled pork with garlic sauce	207.8	79.8	128.0	3.3	8.2	-4.9	20.5	24.8	-4.3	10.1	3.2	6.9
	Black pepper pork foreshank	188.1	171.0	17.1	6.8	10.3	-3.5	19.7	27.8	-8.1	9.5	6.2	3.4
	Poached chicken with scallion oil	142.6	145.0	-2.4	10.7	10.6	0.1	17.5	19.6	-2.1	8.2	7.4	0.8
	Grilled salmon with black sesame seeds	216.2	213.0	3.2	10.7	74.9	-64.2	22.2	28.1	-5.9	9.7	7.6	2.2
	Braised chicken cutlets	142.3	134.0	8.3	5.8	12.9	-7.1	15.4	20.4	-5.0	9.3	6.6	2.7

Table S2. Phosphorus-to-Protein Ratio of Ingredients in the Daily Menu of Low-Phosphate Hospital Diets

Menu	Category	Ingredients	Phosphorus/Protein Ratio (mg/g)
Breakfast menu[a]	Grain	Steamed bread (mantou)	6.9
	Nutritional supplement	LPF, low protein formula (Sentosa Co., Ltd)	15.4
		Whey protein (Sentosa Co., Ltd)	5.5
		MCT, medium-chain triglyceride formula (Sentosa Co., Ltd).	8.5
Lunch or dinner menu[b]	Meat[c]	Grilled lean meat with white sesame seeds	8.8
		Sliced boiled pork with garlic sauce	3.8
		Black pepper foreshank	7.4
		Poached chicken with scallion oil	7.1
		Braised chicken cutlets	7.2
	Side dish	Braised tofu with black fungus	12.7
		Stir-fried cucumber and bean curd skin	13.9
		Stir-fried sweet pepper and bean curd noodles	13.0
		Stir-fried Chinese chive flower and soybean curd	15.3
	Vegetable	Fried sweet potato leaves	11.2
		Fried green bean	17.6
		Fried broccoli	21.1
		Fried pak-choi	20.2
	Grain	Steamed rice	8.1
		Fried bean thread noodles with celery cabbage[d]	29.8
		Fried rice stick noodle with celery cabbage[d]	52.7
	Fruit	Orange	20.7
		Apple	15.8

Note. [a]Breakfast included steamed bread and a mixture of LPF, whey protein and MCT. [b]Lunch or dinner included 1 meat, 1 side dish, 1 vegetable, 1 grain and 1 fruit. [c]The meats had been boiled in water for 30 minutes before cooking to meet low-phosphate requirements. [d]Even though these two foods had a high

phosphorus-to-protein ratio, they were selected to meet food diversity and preferences.

Table S3. Ingredients, Preparation and Cooking Methods of the Study Menu

Food items	Ingredients	Food preparation	Cooking methods
1. Orange	Orange	Peel and core the fruit	None
2. Apple	Fuji apple	Peel and core the fruit	None
3. Steamed bread (mantou)	Steamed bread (mantou)	None	Steaming
4. Stir-fried bean thread noodles with celery cabbage	Bean thread noodles, celery cabbage, soybean oil, soy sauce	Blanch celery cabbage for 30 seconds. Soak bean thread noodles until soft for 10 minutes	Stir-frying
5. Steamed rice	Cooked rice	Wash and drain the rice, then add water to rice at a 1:1.25 ratio of rice:water	Steaming
6. Stir-fried rice stick noodle with celery cabbage	Rice stick noodle, celery cabbage, soybean oil, soy sauce	Blanch celery cabbage for 30 seconds	Stir-frying
7. Roasted pork lean meat with white sesame seeds (A)	Pork lean meat, white sesame seed, soy paste, brown sugar, five spices powder, tapioca, garlic	Marinate pork lean meat with seasonings for 10 minutes, then place a sprinkle of white sesame seeds over the meat	Oven roasting at 135°C for 18 minutes
8. Sliced boiled pork with garlic sauce (A)	Pork lean meat, soy paste, brown sugar, garlic	Slice pork lean meat. Make the garlic sauce with seasonings	Place the meat in hot water until boiling, then drizzle the garlic sauce over the cooked meat
9. Black pepper pork foreshank (A)	Pork foreshank, soy paste, brown sugar, garlic, black pepper grains[2]	Marinate pork foreshank with seasonings for 10 minutes.	Oven roasting at 135°C for 50 minutes
10. Poached chicken with scallion oil (A)	Chicken drumstick[1], green onion[1], carrot[1], tapioca, salt	Season chicken drumsticks with salt and rice-wine	Place chicken drumsticks in a steamer for 30 minutes, cook and thicken the other ingredients with the resultant chicken broth and tapioca, then drizzle the sauce over the cooked chicken

11. Roasted salmon with black sesame seeds (A)	Atlantic salmon (Taiwan aquaculture production), black sesame seed, soy paste, brown sugar, garlic	Marinate salmon with seasonings for 10 minutes	Oven roasting at 135°C for 20 minutes
12. Braised chicken cutlets (A)	Chicken cutlets[1], soy sauce, brown sugar, garlic, soybean oil	Make the marinade with seasonings	Stew chicken cutlets with marinade for 30 minutes
13. Roasted pork lean meat with white sesame seeds (B)	Pork lean meat, white sesame seed, soy paste, brown sugar, five spice powder, tapioca, garlic	Blanch the pork lean meat, then immerse the meat in a pot with boiling water using a steam oven at 100°C for 30 minutes. Marinate the meat with seasonings for 10 minutes, then place a sprinkle of white sesame seeds over meat	Oven roasting at 135°C for 15 minutes
14. Sliced boiled pork with garlic sauce (B)	Pork lean meat, soy paste, brown sugar, garlic	Slice pork lean meat, then immerse the meat in a pot with boiling water using a steam oven at 100°C for 30 minutes. Make the garlic sauce with seasonings	Place the meat in hot water until boiling, then drizzle the garlic sauce over the cooked meat
15. Black pepper pork foreshank (B)	Pork foreshank, soy paste, brown sugar, garlic, black pepper grains[2]	Blanch the pork foreshank, then immerse the meat in a pot with boiling water using a steam oven at 100°C for 30 minutes. Marinate the meat with seasonings for 10 minutes	Oven roasting at 135°C for 15 minutes
16. Poached chicken with scallion oil (B)	Chicken drumstick[1], green onion[1], carrot[1], tapioca, salt	Blanch the chicken drumsticks, then immerse the meat in a pot with boiling water using a steam oven at 100°C for 30 minutes. Season the meat with salt and rice-wine	Place chicken drumsticks in a steamer for 30 minutes, cook and thicken the other ingredients with the resultant chicken broth and tapioca, then drizzle the sauce over the cooked chicken

17. Roasted salmon with black sesame seeds (B)	Atlantic salmon (Taiwan aquaculture production), black sesame seed, soy paste, brown sugar, garlic	Blanch the salmon, then immerse the meat in a pot with boiling water using a steam oven at 100°C for 30 minutes. Marinate the meat with seasonings for 10 minutes	Oven roasting at 135°C for 15 minutes
18. Braised chicken cutlets (B)	Chicken cutlets[1], soy sauce, brown sugar, garlic, soybean oil	Blanch the chicken cutlets, then immerse the meat in a pot with boiling water using a steam oven at 100°C for 30 minutes. Make the marinade with seasonings	Stew chicken cutlets with marinade for 20 minutes
19. Braised tofu with black fungus	Tofu, carrot[1], black fungus, soybean oil, soy paste, brown sugar	Blanch the ingredients individually	Stir-frying
20. Stir-fried cucumber and bean curd skin	Bean curd skin, cucumber, carrot[1], soybean oil, soy paste, brown sugar	Blanch the ingredients individually	Stir-frying
21. Stir-fried sweet pepper and bean curd noodles	Bean curd noodles, sweet pepper[1], soybean oil, soy paste	Blanch the ingredients individually	Stir-frying
22. Stir-fried Chinese chive flower and soybean curd	Soybean curd, Chinese chive flower, carrot[1], soybean oil, soy paste	Blanch the ingredients individually	Stir-frying
23. Stir-fried sweet potato leaves	Sweet potato leaves, soybean oil, salt	Blanch the ingredients individually	Stir-frying
24. Stir-fried green bean	Green bean, soybean oil, salt	Blanch the ingredients individually	Stir-frying
25. Stir-fried broccoli	Broccoli, soybean oil, salt	Blanch the ingredients individually	Stir-frying
26. Stir-fried pak-choi	Pak-choi[1], soybean oil, salt	Blanch the ingredients individually	Stir-frying

Note. [1]Average values of nutrients in the food composition database were used. [2]The estimation of nutrients for black pepper grains was replaced by red pepper grains due to lack of information in the nutrient database. "(A)" means meats are cooked without extra boiling for 30 minutes and "(B)" means meats are boiled in water for 30 minutes before cooking.
